# Low Mate Encounter Rate Increases Male Risk Taking in a Sexually Cannibalistic Praying Mantis

**DOI:** 10.1371/journal.pone.0035377

**Published:** 2012-04-25

**Authors:** William D. Brown, Gregory A. Muntz, Alexander J. Ladowski

**Affiliations:** Department of Biology, State University of New York at Fredonia, Fredonia, New York, United States of America; University of Western Ontario, Canada

## Abstract

Male praying mantises are forced into the ultimate trade-off of mating versus complete loss of future reproduction if they fall prey to a female. The balance of this trade-off will depend both on (1) the level of predatory risk imposed by females and (2) the frequency of mating opportunities for males. We report the results of a set of experiments that examine the effects of these two variables on male risk-taking behavior and the frequency of sexual cannibalism in the praying mantis *Tenodera sinensis*. We experimentally altered the rate at which males encountered females and measured male approach and courtship behavior under conditions of high and low risk of being attacked by females. We show that male risk taking depends on prior access to females. Males with restricted access to females showed greater risk-taking behavior. When males were given daily female encounters, they responded to greater female-imposed risk by slowing their rate of approach and remained a greater distance from a potential mate. In contrast, males without recent access to mates were greater risk-takers; they approached females more rapidly and to closer proximity, regardless of risk. In a second experiment, we altered male encounter rate with females and measured rates of sexual cannibalism when paired with hungry or well-fed females. Greater risk-taking behavior by males with low mate encounter rates resulted in high rates of sexual cannibalism when these males were paired with hungry females.

## Introduction

Praying mantises are famously associated with sexual cannibalism – the consumption of a male by his mate before, during or after sex [1], [2]. In reality, the occurrence of sexual cannibalism in mantises is highly variable among species [3], with upper estimates of about 46% of mating attempts for the Australian *Pseudomantis albofimbriata* [4] and other species, such as members of the genus *Ciulfina*, lacking sexual cannibalism altogether [5]. In another well-known case of sexual cannibalism, the Australian redback spider, males often appear to be willing victims, voluntarily somersaulting into the jaws of their mate and thereby achieving longer copulation duration, greater sperm transfer and greater paternity of her offspring [6]. The male of the Chinese mantis *Tenodera sinensis*, in contrast, is no such willing victim. Mate attraction is guided by airborne pheromones produced by females [7]–[10]. Males typically approach females with great stealth and care, slowing down, staying farther away, and courting more vigorously when females are hungry and when males must approach head-on, into the predatory “attack zone” of the female [11]. Cannibalism of male mantises by females is thus a prime example of sexual conflict; males are forced into the ultimate trade-off of mating versus complete loss of future reproduction if they fall prey to a female. The balance of this trade-off will depend both on the level of predatory risk imposed by females and on the frequency of mating opportunities for males [12]–[14].

Barry and Kokko [14] recently developed a model of male choice for species, such as mantises, that have extreme differences in the value of different mating attempts caused by risk of sexual cannibalism. Their model predicts that males will evolve to be more discriminating of females when there is a high overall cost of approaching a female, owing to a high rate of cannibalism, and will largely depend upon (1) the proportion of risky females and (2) the frequency of mate encounters. Here, we report the results of a set of experiments that examine the effects of these two variables on male risk-taking behavior and the frequency of sexual cannibalism in the praying mantis *T. sinensis*. We use an experimental design in which individual males are tested under both (a) high and low risk conditions and (b) after periods of both high and low access to potential mates. Thus differences in male response to risky females may be influenced by both perceived availability of females and by learned responses to female aggression. We show that males with restricted access to females show greater risk-taking behavior and a higher incidence of sexual cannibalism when presented to especially dangerous females.

## Materials and Methods

### Ethics Statement

No specific permits were required for the described studies. Permission to access the location for field studies was provided by the landowner, J. L. Berkley. We confirm that the study did not involve endangered or protected species.

### Study population

We captured mantises as adults and final-instar juveniles from wild populations in Chautauqua Co., NY, USA. Note that, according to Jensen et al. [15], populations in the USA belong to the species *T. sinensis* and not *T. aridifolia sinensis* as we had indicated in previous publications [8], [11]. All mantises used in experimental trials were collected in late August and early September, prior to outset of the breeding season. Late instar juveniles were reared until they emerged as adults and maintained for at least 1 week prior to being used in the experiments. Mantises were housed as described previously [8], [11]. Briefly, we placed individual mantises into 500 ml plastic terrariums and provided *ad libitum* crickets, *Acheta domesticus*, as food. Mantises were maintained at 22°C and a 12∶12 light cycle.

### Male behavior in response to risk and encounter rate

We experimentally altered male access to females and compared male approach and courtship under low- and high-risk conditions. Treatments included (a) high or low prior access to females, and (b) high or low female-imposed risk of attack. Males in the low-access treatment were maintained individually in 1-l plastic cages and isolated from all contact with females for 5 d prior to testing behavior. Males with high access to females were allowed periods of daily, unrestricted one-on-one contact with females for 5 d (high access to females). We placed these males together with a single, adult female in a 20-l glass terrarium. To reduce the occurrence of sexual cannibalism, the females were fed crickets ad libitum prior to their encounters with males. Pairs that did not mate were separated after 1 h. Pairs that mated during this period remained together until their genitalia uncoupled (copulation duration averages 207 min in *T. sinensis* [11]). We then returned males and females to their individual 1-l plastic cages. Males in the high-access treatment encounter a different female each day. Males on the high-access treatment mated an average of 2.37±0.25 (SE) times prior to testing. All males received a diet of *ad libitum* crickets, *A. domesticus*.

Males were tested in response to two treatments that alter female-imposed risk of cannibalism. Note that our intention was to maximize or minimize male perception of risk and thus risk is the combined effect of female hunger and orientation. We do not attempt to distinguish the components of risk (hunger versus orientation); this was part of a previous study [11]. Under high-risk conditions we starved females for 5 d. Prior studies have shown that hungry female mantids are significantly more rapacious than satiated females [16]–[19] and males recognize the hunger status of females [11]. We then placed a female at the end of a wooden plank (80 cm long×6 cm wide) and oriented her to face toward the male, thus forcing the male to approach into the predatory “attack zone” of a hungry female [20]. Under low-risk conditions, we satiated females by feeding them crickets, *A. domesticus*, ad libitum prior to the experiment. We oriented these females facing away from the male at the end of the wooden plank to allow the male to approach from behind, away from the females' predatory forelimbs. Each male was randomly paired with a female and this pair was tested under the four treatment combinations, in random order. There was a minimum of 6 d between trials of a given pair as required to alter female hunger levels and male access to mates.

We recorded male approach speed, distance between male and female prior to mounting, and courtship as described previously [11]. Approach speed was measured as the distance that the male traveled on foot toward the female divided by the time taken to travel this distance. We measured distance prior to mounting as the distance between the male and female from which the male leapt onto the back of the female to mount. Courtship in *T. sinensis* takes the form of a rhythmic bending motion of the abdomen [17] and we measured courtship intensity as the angle of abdominal bending by the male [11]. We terminated trials immediately upon an aggressive strike at the male by the female to avoid cannibalism and allow males to be tested under multiple treatment combinations. We tested 27 mantis pairs for a total of 90 trials (3.33±0.18 trials/pair). We were unable to achieve all treatment combinations for all pairs owing to (non-cannibalism) mortality between trials. In 8 cases, males did not mount females and these are excluded from analysis of distance prior to mounting.

### Sexual cannibalism in response to risk and encounter rate

In the second experiment, prior exposure to females and female hunger (and thus risk of cannibalism) were manipulated as in the first experiment. We did not however control orientation of the female to the male. Mantises were assigned randomly to treatments and each mantis (N = 74 pairs) was used only once. We first placed a female on the bottom of a 30 cm cubic cage constructed of fine mesh (ca. 1.2 mm), polyester netting on an aluminum frame (BioQuip, Gardena, CA, USA). We then placed the male in the top corner of the cage ca. 30–40 cm from the female. We used scan sampling every 10 min to record latencies to mounting, mating, and/or sexual cannibalism. Each trial ended either when (a) the male dismounted after mating, (b) the female finished cannibalizing the male, or (c) after 360 min., whichever happened first.

### Statistical analyses

We analyzed the data from the first experiment on male behavior using linear mixed models (LMM). LMM extends general linear models (GLM) to better support repeated measures and random effects [21]. We incorporated mantis ID as a repeated subject variable. Approach speed was normalized by natural-log transformation prior to analysis. For the second experiment on male survival rates under risk of cannibalism, we performed survival analysis using a Cox regression with right-censored data.

## Results

### Male behavior in response to risk and encounter rate

**Figure 1:**
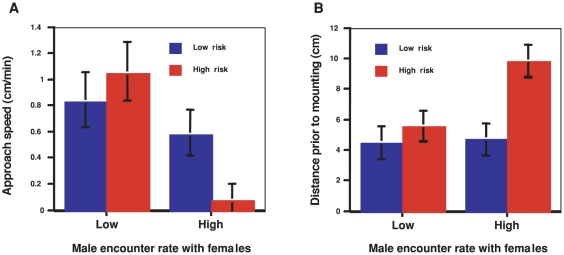
Male approach toward females. (**A**) Males with high female encounter rate approached high-risk females more slowly than they approached low-risk females, whereas males with low female encounter rate showed no difference in approach rate. (**B**) Males with high access to females approached remained farther away from high-risk females but males with low encounter rate approached equally closely to low- and high-risk females.

High-risk females made significantly more aggressive strikes at males (Wald = 15.58, p<0.001). We hypothesized that males with low prior access to females would be less discriminate and exhibit greater overall risk taking behavior, compared to males with higher access to mates. Thus we explicitly predicted significant treatment interactions, with high access males displaying greater differences in behavior in response to female-imposed risk. As predicted, male approach speed demonstrated a significant treatment interaction (F_1,65_ = 6.82, p = 0.01); males with low access approached females relatively quickly (Figure 1A), prior to mounting. Distance prior to mounting also showed a significant treatment interaction (F_1,57_ = 3.86, p<0.05) (Figure 1B); males with high access to females remained farther away from high-risk females to prior to mounting.

There were also significant independent effects of male access to females and female-imposed risk of attack. Males with low access to females approached females more quickly overall (F_1,65_ = 17.43, p<0.001) and more closely (i.e., lower distance prior to mounting) (F_1,57_ = 4.77, p = 0.03), compared to males with high access to mates.

Risk treatment had no significant independent effect on male approach speed (F_1,57_ = 1.97, p = 0.17), but males remained significantly farther away from the risky females prior to mounting (F_1,57_ = 8.97, p = 0.004) (Figure 1B). As males approached these high-risk females, they engaged in significantly more elaborate courtship (abdominal bends, measured as the angle from the long axis of the body) (F_1,65_ = 4.64, p = 0.03). There was no significant effect of male access to females on courtship behavior (F_1,65_ = 2.01, p = 0.16), nor was there a significant treatment interaction (F_1,65_ = 0.005, p = 0.94).

Thus high-access males appeared to differentiate among females based on risk, whereas low-access males demonstrated behavior indicating greater overall risk taking. Males with low prior access to females made faster and closer approaches regardless of the level of female-imposed risk of attack. In contrast, males with high prior access to females reacted strongly to female-imposed risk by reducing their rate of approach (Figure 1A), and increasing their distance (Figure 1B) from high-risk females.

### Sexual cannibalism in response to risk and encounter rate

**Figure 2:**
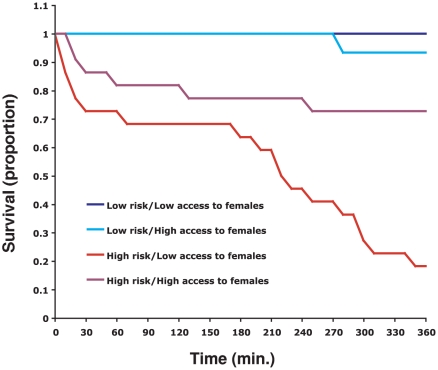
Survivorship curves for males for males for with low access or high prior access to females and under low- or high-risk of being attacked by female. Compared to males with high access to females, males with low access to females experienced a greater decrease in survival caused by a higher rate of sexual cannibalism when present to hungry females.

In a second experiment we examined the effects of male access to females and female hunger on rates of sexual cannibalism. We placed single males that had been given either high or low prior access to females together with single starved or ad libitum fed female mantises and recorded occurrences of sexual cannibalism over 6 h. Compared to males with high access to females, males with low access to females experienced a greater decrease in survival when presented to hungry females, but not when presented to well-fed females (Cox regression: Wald = 4.59, p = 0.03). Thus the lowest male survival (and hence the greatest incidence of sexual cannibalism) occurred when low-access males faced hungry females (Figure 2). Neither female hunger (Wald = 0.48, p = 0.49) nor male access to females (Wald = 0.02, p = 0.88) had significant independent effects on male survival.

## Discussion

Male mantises with restricted access to females are bigger risk-takers. Males given no interactions with females for 5 d showed no evidence of discriminating between females based on hunger and orientation, other than displaying greater courtship when facing hungry females head on. In contrast, males that experienced daily encounters with females prior to testing significantly slowed their rate of approach, and stayed farther away, when approaching risky females head on. Males thus appear to assess variation in the risk imposed by females and become more cautious in their approach of females that are more likely to attack [11]. However, as predicted by the model of Barry and Kokko [14] the level of male discrimination of females appears to depend on the frequency of mate encounters, with low encounter rates generating less discrimination and greater overall risk taking by males.

In our experiment, this difference in male discrimination of females could be due to greater motivation to mate regardless of risk, given the rarity of past encounters. That is, whereas males assess and respond to female-imposed risk by approaching risky females with greater caution when females are common, males may have a history of selection to become indiscriminate, and fully exploit any potential mating opportunity, when females are rare [12], [14]. Alternatively, the difference in male discrimination of females could be a learned response from past experience with females. If males learn to assess risk and avoid attacks during active encounters with females, then these experiences over 5 d prior to testing may facilitate greater risk-avoidance behavior by high access males. However, our experiment allowed half the males to experience high female encounter rate prior to being isolated from females for 5 d. Thus this second alternative would require that males forget or ignore experiences of greater than 5 d prior to the treatment. Distinguishing among these alternatives is a possible avenue of future research.

Our second experiment connects female imposed risk and male access to females directly with episodes of sexual cannibalism. Ad libitum fed females exhibited very low rates of sexual cannibalism, with only one case overall (when encountering a high-access male). Frequency of sexual cannibalism was much greater when males encountered hungry females [16]–[19]. Rates of sexual cannibalism were greater than 20% over 360 min for high-access males facing hungry females, similar to estimated rates of cannibalism in field populations of *T. sinensis* [22]. This rate rose to nearly 80% for low-access males facing hungry females. This last number represents among the highest frequency of sexual cannibalism reported for mantises [16]. Although this high frequency of sexual cannibalism may be facilitated by the experiment set up (which may limit male ability to escape), the strong differences among treatments suggest that slow, cautious approach of high-risk females by high-access males has real consequences for reducing mortality resulting from sexual cannibalism.
